# Sex-Specificity of Mineralocorticoid Target Gene Expression during Renal Development, and Long-Term Consequences

**DOI:** 10.3390/ijms18020457

**Published:** 2017-02-21

**Authors:** Laurence Dumeige, Caroline Storey, Lyvianne Decourtye, Melanie Nehlich, Christophe Lhadj, Say Viengchareun, Laurent Kappeler, Marc Lombès, Laetitia Martinerie

**Affiliations:** 1 Inserm U1185, Univ Paris Sud, Université Paris-Saclay, F-94276 Le Kremlin-Bicêtre, France; ldumeige@u-psud.fr (L.D.); caroline.storey@aphp.fr (C.S.); melanie.nehlich@yahoo.fr (M.N.); christophe.lhadj@u-psud.fr (C.L.); say.viengchareun@u-psud.fr (S.V.); laetitia.martinerie@aphp.fr (L.M.); 2 Service d’Endocrinologie Pédiatrique, Hôpital Robert Debré, Assistance Publique Hôpitaux de Paris, F-75019 Paris, France; 3 Faculté de Médecine Paris Diderot, Université Paris Diderot, Sorbonne Paris Cité, F-75019 Paris, France; 4 Inserm, UMR_S938, Centre de Recherche Saint-Antoine, F-75012 Paris, France; lyvianne.decourtye@inserm.fr (L.D.); laurent.kappeler@inserm.fr (L.K.); 5 PremUp Foundation, F-75005 Paris, France; 6 Faculté de Médecine Paris-Sud, UMR-S1185, Université Paris-Sud 11, F-94276 Le Kremlin-Bicêtre, France; 7 Service d’Endocrinologie et Maladies de la Reproduction, Hôpital de Bicêtre, Assistance Publique-Hôpitaux de Paris, F-94275 Le Kremlin Bicêtre, France

**Keywords:** mineralocorticoid signaling pathway, sexual dimorphism, gene expression, hypertension

## Abstract

Sex differences have been identified in various biological processes, including hypertension. The mineralocorticoid signaling pathway is an important contributor to early arterial hypertension, however its sex-specific expression has been scarcely studied, particularly with respect to the kidney. Basal systolic blood pressure (SBP) and heart rate (HR) were measured in adult male and female mice. Renal gene expression studies of major players of mineralocorticoid signaling were performed at different developmental stages in male and female mice using reverse transcription quantitative PCR (RT-qPCR), and were compared to those of the same genes in the lung, another mineralocorticoid epithelial target tissue that regulates ion exchange and electrolyte balance. The role of sex hormones in the regulation of these genes was also investigated in differentiated KC3AC1 renal cells. Additionally, renal expression of the 11 β-hydroxysteroid dehydrogenase type 2 (11βHSD2) protein, a regulator of mineralocorticoid specificity, was measured by immunoblotting and its activity was indirectly assessed in the plasma using liquid-chromatography coupled to mass spectrometry in tandem (LC-MSMS) method. SBP and HR were found to be significantly lower in females compared to males. This was accompanied by a sex- and tissue-specific expression profile throughout renal development of the mineralocorticoid target genes serum and glucocorticoid-regulated kinase 1 (*Sgk1*) and glucocorticoid-induced leucine zipper protein (*Gilz*), together with *Hsd11b2*, Finally, the implication of sex hormones in this sex-specific expression profile was demonstrated in vitro, most notably for *Gilz* mRNA expression. We demonstrate a tissue-specific, sex-dependent and developmentally-regulated pattern of expression of the mineralocorticoid pathway that could have important implications in physiology and pathology.

## 1. Introduction

Sex differences in biological and physiological characteristics have been demonstrated with chromosomes, reproductive organs and hormonal biosynthesis, secretion and action. They have also recently been shown in various biological processes such as white matter development and brain volume [1], stress response and depression [2] or respiratory function [3].

Arterial blood pressure is one of these processes. Indeed, epidemiological studies of cardiovascular diseases in men and women as well as in experimental animal models show substantial sexual dimorphism in the incidence and prevalence of arterial hypertension [4,5,6,7]. The renin–angiotensin–aldosterone system and, by extension, the mineralocorticoid receptor (MR) signaling pathway is an important contributor to the emergence of early arterial hypertension and heart failure, with clinical studies demonstrating the beneficial impact of the use of anti-mineralocorticoid drugs [8,9,10]. Moreover, evidence supports the central role of the kidney in the control of blood pressure via the regulation of sodium homeostasis and glucocorticoid metabolism [11,12].

The mineralocorticoid pathway is regulated by aldosterone, a steroid hormone, synthesized by the adrenal cortex, whose principal role concerns sodium homeostasis. This has been well documented in both humans and animals. Aldosterone stimulates sodium reabsorption and potassium excretion in the distal parts of the nephron, thus participating in the control of plasma volume and blood pressure. This is particularly relevant for different pathologies associated with aldosterone excess such as primary hyperaldosteronism and genetic-familial hyperaldosteronisms [13]. The main biological effects of aldosterone at the cellular level are mediated through genomic and non-genomic pathways. The classical, well-described genomic pathway involves binding to the specific MR (*Nr3c2*), a transcription factor which undergoes hormone-dependent nuclear translocation upon aldosterone binding. The MR then dimerizes and binds specific hormone response elements on DNA [14], leading to the transactivation of various target genes implicated in epithelial tissues in the reabsorption of sodium, such as the α subunit of the epithelial sodium channel (αENaC, *Scnn1a*), the serum and glucocorticoid-regulated kinase 1 (*Sgk1*) and the glucocorticoid-induced leucine zipper protein (*Gilz*) [15]. These target genes are also activated by glucocorticoids and the glucocorticoid signaling pathway, mediated by the glucocorticoid receptor (GR), which shares hormone response elements and co-activators with the mineralocorticoid receptor [15]. Aldosterone and glucocorticoids can bind MRs with the same affinity. Thus, mineralocorticoid selectivity is ensured in epithelial target cells by the 11 β-hydroxysteroid dehydrogenase type 2 (11βHSD2) enzyme [16,17], which converts cortisol (or corticosterone in rodents) into inactive compounds : cortisone (or 11-dehydrocorticosterone in rodents).

While MR expression is well established in physiology and disease during development and in adults [15,18], its potential sex-specific expression pattern has been rarely studied [6,19,20,21]. Particularly, it has been suggested that the estrogen receptor could exert antagonistic effects on the mineralocorticoid pathway via its dimerization with the MRs in epithelial cells, which could participate in the prevention of women from cardiovascular diseases before menopause [21]. The aim of the present study was to further investigate whether there are sex differences in regulation of MR signaling outcomes and to demonstrate the existence of a sex-specific pattern of expression of different players of the mineralocorticoid receptor pathway. It was anticipated to possibly define a relationship between these results and variations in basal arterial blood pressure and heart rate in male and female wild-type mice.

## 2. Results

### 2.1. Sexual Dimorphism of Systemic Blood Pressure and Heart Rate in Mice

**Figure 1:**
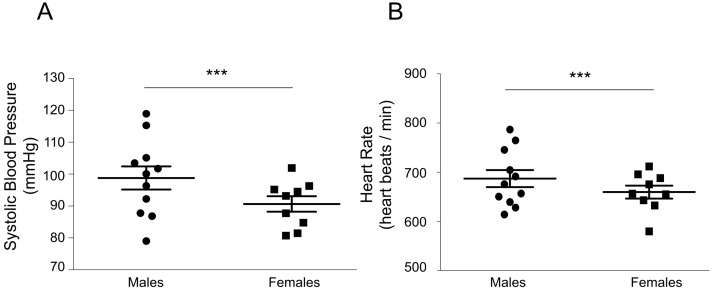
Sexual dimorphism in basal systolic blood pressure (**A**); and heart rate (HR) (**B**) in adult mice. Blood pressure measurements were conducted in the animal facility of the FRIM (Fédération de Recherche en Imagerie Multi-Modalité, Paris Diderot University, Paris, France). Nine female mice and eleven male mice aged 6 months were used for the analysis. Results are expressed as dots which represent the mean of at least six measures of systolic blood pressure and HR for each animal and bars represent the mean ± SEM of all female or male measurements. *** *p* < 0.001.

Basal systolic blood pressure and heart rate (HR) were measured in male (*n* = 11) and female (*n* = 9) 6-month-old mice originating from a mixed genetic background (B6D2 F1) to minimize any specific strain effect. Results were obtained from two independent experiments (Figure 1). Mean systolic blood pressure was significantly lower in females than in males (91.7 ± 1.1 vs. 99.2 ± 1.0 mmHg, *p* < 0.0001). Similarly, HR (expressed as heart beats per min, bpm) was also significantly lower in females than in males (662 ± 3 vs. 687 ± 4 bpm, *p* < 0.0001), confirming the sexually dimorphic pattern of these two parameters.

### 2.2. Sexual Dimorphism in the Expression of Target Genes of the Mineralocorticoid Receptor Signaling Pathway during Renal Development

**Figure 2:**
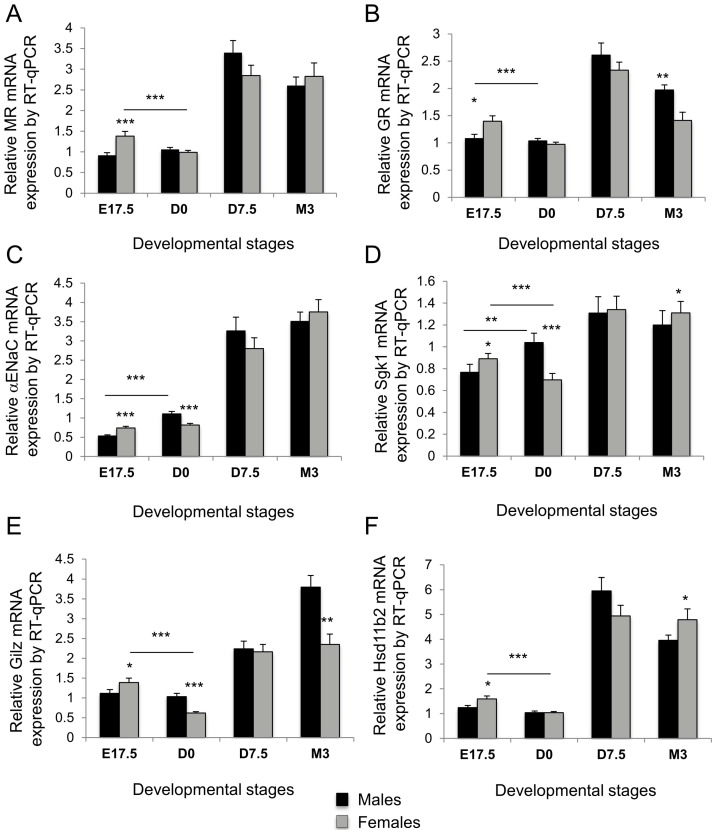
Sexual dimorphism in mineralocorticoid receptors (MRs), glucocorticoid receptor (GRs), *Hsd11b2* and several MR-regulated target gene expressions during renal development in mice. MRs (**A**); GRs (**B**); α subunit of the epithelial sodium channel (*αENaC*) (**C**); serum and glucocorticoid-regulated kinase 1 (*Sgk1*) (**D**); glucocorticoid-induced leucine zipper protein (*Gilz*) (**E**); and *Hsd11b2* (**F**) renal relative mRNA expressions throughout development in mice were determined using reverse transcription quantitative PCR (RT-qPCR) at various developmental stages as follows: 17.5 days of gestation (E17.5), day of birth (D0), D7.5 and M3. E: Embryonic day, D: postnatal day, M: postnatal month. Results, expressed as the ratio of attomoles of specific gene per femtomoles of ribosomal 18S, normalized to the expression of male mice at D0, arbitrarily set at 1, correspond to mean ± SEM of three independent experiments comprising at least 6 male and 6 female kidneys each. * *p* < 0.05, ** *p* < 0.01, *** *p* < 0.001. Note that statistical significances between different developmental stages from D0 to D7.5 are not represented on the graph for better clarity, but for all *p* < 0.0001.

**Figure 3:**
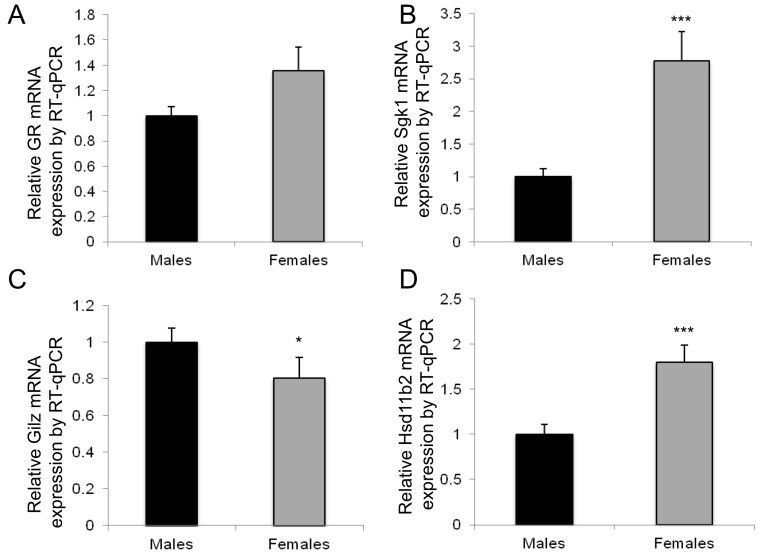
Sexual dimorphism in renal MRs, GRs, *Hsd11b2* and several MR-regulated target gene expressions in adult (M3) mice in another independent series of 12 animals (6 males and 6 females) of a different strain (C57B6/129S). GRs (**A**); *Sgk1* (**B**); *Gilz* (**C**); and *Hsd11b2* (**D**) renal relative mRNA expressions in adult mice (M3: 3 months of age) were determined using RT-qPCR. Results, expressed as the ratio of attomoles of specific gene per femtomole of ribosomal 18S, normalized to the expression of male mice and arbitrarily set at 1, correspond to mean ± SEM of two independent reverse-transcriptions comprising at least 6 male and 6 female kidneys each. * *p* < 0.05, *** *p* < 0.001.

Given that blood pressure and HR are at least partially dependent on the regulation and activation of the aldosterone–MR mediated processes, we next investigated whether this sexual dimorphism could be somehow associated with variations in the expression of specific renal MR-regulated target genes. We also evaluated if these sex-specific differences emerged only in adulthood or were readily present during renal development (Figure 2). We thus analyzed the expression of MRs and GRs during renal development, and also that of several target genes, α*ENaC*, *Sgk1* and *Gilz*, as well as that of the *Hsd11b2*, which confers mineralocorticoid selectivity. Results represent the relative expression compared to that obtained in samples from male mice at postnatal day 0 (D0), arbitrarily set at 1. As previously demonstrated [18], these different MR-regulated target genes had a low expression in the kidney at birth and their expression rose significantly in the postnatal period. This was confirmed in the present experiments both for male and female mice (*p* < 0.0001). However, when comparing males and females, and focusing on the perinatal period, it appeared that in females there is an antenatal peak of renal expression at 17.5 days of gestation (E17.5) for most of these genes, with significant downregulation at birth (*p* < 0.0001), while in males the expression is steady (for MRs, GRs, *Hsd11b2*, *Gilz*) or rises significantly from E17.5 to D0 (αENaC and *Sgk1*, *p* < 0.001 and *p* < 0.0001, respectively), thus emphasizing an early sexual dimorphism expression pattern of these genes in the kidney. This sexual dimorphism persists in adulthood, as 3-month-old female mice displayed a significant difference compared to males of the same age, in the expression of *Gr* (*Nr3c1*) and *Gilz* (significantly lower, *p* < 0.01) and *Hsd11b2* and *Sgk1* (significantly higher, *p* < 0.05). These results were confirmed in another series of experiments performed in 3-month-old animals of mixed genetic background (C57B6/129S, Figure 3). Renal expression of *Sgk1* and *Hsd11b2* are 2- to 2.5-fold higher in females compared to males, while *Gilz* mRNA level is 2-fold lower in females than in males. In these experiments, mean body weight differed between males and females (mean ± SEM: 30.5 ± 0.3 g, *n* = 12, vs. 26.1 ± 0.4 g, *n* = 10, *p* < 0.001, however the ratio of kidney weight/total body weight was similar in each group, *p* = 0.15). Thus, this renal sexual dimorphism in gene expression is conserved between various mouse strains, emphasizing its potential and well-conserved physiological importance.

**Figure 4:**
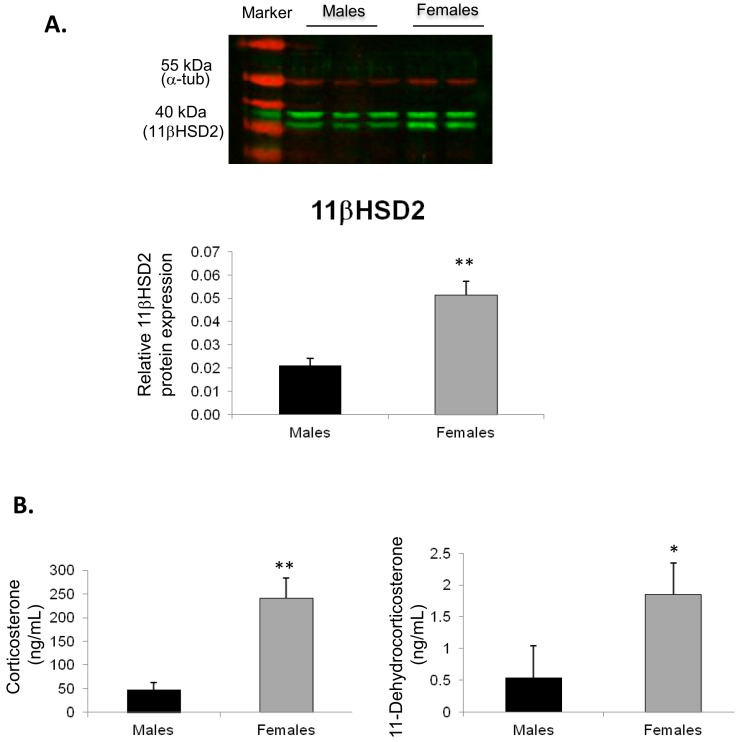
(**A**) Comparison of 11 β-hydroxysteroid dehydrogenase type 2 (11βHSD2) renal protein expression between male and female adult mice. The relative protein expression of 11βHSD2 was quantified using immunoblot analyses in kidneys of 6 adult male and 5 adult female mice. Note that the 11βHSD2 antibody leads to the detection of two bands of 45 and 40 kDa molecular masses, the lower band corresponding to the 11βHSD2 protein (**upper panel**). Results, expressed as the ratio of the expression of the 11βHSD2 protein, normalized to that of the α-tubulin used as loading control, correspond to mean ± SEM (**lower panel***)*, ** *p* < 0.01; (**B**) Comparison between corticosterone and 11-dehydrocorticosterone plasma levels between male and female adult mice. These hormonal levels were measured by liquid-chromatography coupled to mass spectrometry in tandem (LC-MSMS) in 6 adult male and 5 adult female mice. Results represent mean ± SEM. * *p* < 0.05, ** *p* < 0.01.

To evaluate whether these expressions were also modified in a sexually dimorphic manner at the protein level, we performed immunoblot analyses using the specific polyclonal anti-mouse 11βHSD2 antibody in two series of 3-month-old male and female kidneys. As shown in Figure 4A, immunoblotting revealed two bands migrating at 45 and 40 kDa molecular masses, the lower band likely corresponding to 11βHSD2 protein. A sex-specific, statistically significant, differential 11βHSD2 protein expression was observed, with an increased expression of this protein in females compared to males, corroborating our reverse transcription quantitative PCR (RT-qPCR) results. Of note, the commercially available anti-Sgk1 and anti-Gilz antibodies do not work with whole kidney extracts and thus were not useful in our studies. Furthermore, using the liquid-chromatography coupled to mass spectrometry in tandem (LC-MSMS) method, we quantified corticosterone and 11-dehydrocorticosterone plasma levels in 6 males and 5 females (Figure 4B). Hormonal levels were significantly higher in females compared to males (1.85 ± 0.39 ng/mL vs. 0.54 ± 0.18 ng/mL for 11-dehydrocorticosterone, *p* = 0.0016, and 257.8 ± 32.2 vs. 47.2 ± 15.6 ng/mL for corticosterone, *p* = 0.0004, respectively), with a ratio between these two compounds which estimates the equilibrium between both 11βHSD2 and 11βHSD1 activities that was statistically different between both sexes (*p* = 0.012). In addition, aldosterone levels were also measured by LC-MSMS, however no difference was observed between males and females (0.09 ± 0.02 pg/mL vs. 0.07 ± 0.03 pg/mL, *p* = 0.63).

### 2.3. Tissue Specificity of Sexual Dimorphic Gene Expression of MRs, GRs, Hsd11b2 and MR-Regulated Target Genes

**Figure 5:**
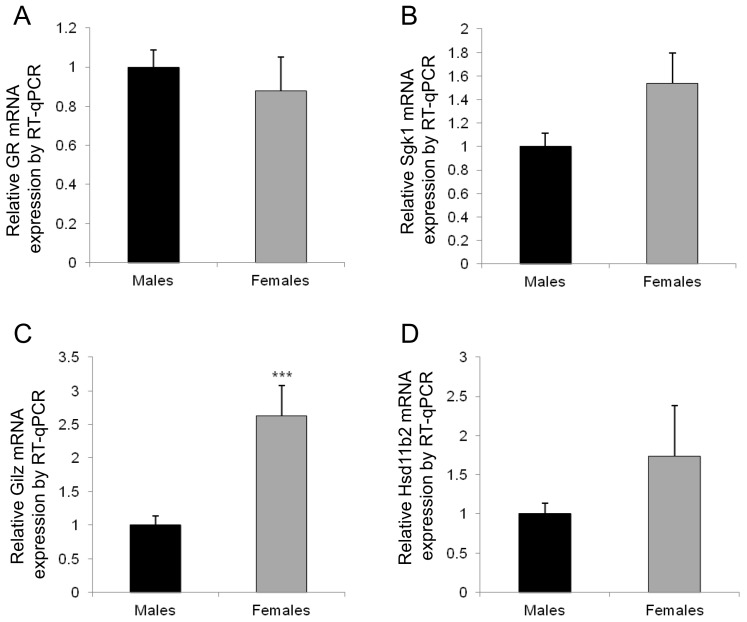
Tissue-specificity in sexual dimorphism in the expression of GRs, *Hsd11b2* and several MR-regulated target genes: example in the lung of adult (M3) mice. GR (**A**); *Sgk1* (**B**); *Gilz* (**C**); and *Hsd11b2* (**D**) pulmonary relative mRNA expressions in adult mice (M3: 3-month-old) were determined using RT-qPCR. Results, expressed as the ratio of attomoles of specific gene per femtomole of ribosomal 18S, normalized to the expression of male mice arbitrarily set at 1, correspond to mean ± SEM of three independent experiments comprising at least 6 male and 6 female lungs each. *** *p* < 0.001.

We next investigated whether the sex-specificity in the pattern of expression of these different actors of the mineralocorticoid signaling pathway was specific to the kidney or if tissue specificity existed as already had been demonstrated for its global expression throughout development, with striking differences between tissues like those of the kidney and the lung [22]. Lungs, collected from the same 3-month-old male and female mice as above, were processed for analysis of mRNA expression of the same genes (Figure 5). As a result, a quite different profile was observed. Indeed, while *GR*, *Hsd11b2* and *Sgk*1 mRNA levels did not show statistical difference in their pulmonary expression among sex, *Gilz* expression in the lung displayed a significant difference between males and females as its expression was approximately 2.5-fold higher in females than in males *(p* < 0.0001), at variance with the kidney where renal *Gilz* expression was lower in females than in males (Figure 5). Collectively, the mineralocorticoid signaling pathway does exhibit a sexual dimorphic and tissue-specific expression pattern during development and in adulthood.

### 2.4. Sex Hormone Influence on the Expression of MRs, GRs and MR-Regulated Target Genes In Vitro

**Figure 6:**
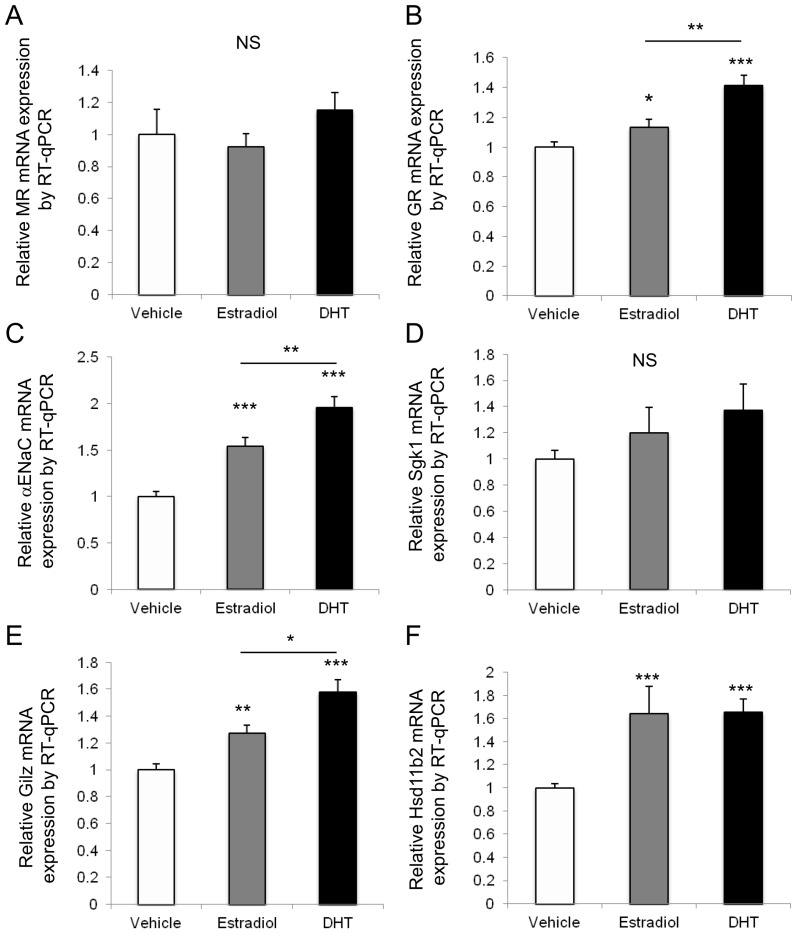
Sex hormone (estradiol and dihydrotestosterone, DHT) influence on the expression of MRs, GRs, *Hsd11b2* and several MR-regulated target genes in vitro (in KC3AC1 renal tubular differentiated cells). MR (**A**); GR (**B**); *αENaC* (**C**); *Sgk1* (**D**); *Gilz* (**E**); and *Hsd11b2* (**F**) relative mRNA expressions in KC3AC1 cells were determined using RT-qPCR, under various conditions: after 24 h of culture with either ethanol (vehicle), 10^−7^ M estradiol or 10^−7^ M DHT. Results, expressed as the ratio of attomoles of specific gene per attomoles of housekeeping gene, *36B4*, normalized to the expression obtained in cells incubated with vehicle arbitrarily set at 1, correspond to mean ± SEM of three independent reverse-transcriptions after RNA extraction of eight independent wells for each condition. * *p* < 0.05, ** *p* < 0.01, *** *p* < 0.001. NS: Not Significant.

To test whether differences in gene expression observed between males and females were directly linked to sex hormone secretion and action, we evaluated the impact of estradiol and dihydrotestosterone in vitro. For this purpose, we used the well-characterized KC3AC1 renal tubular cell line [23], that expresses most of the genes involved in the mineralocorticoid signaling pathway. KC3AC1 cells were cultivated for 7 days in complete medium, then cells were incubated for 24 h in minimum medium. Thereafter, cells were incubated for 24 h either with vehicle (ethanol) as a control, or with 10^−7^ M estradiol, or with 10^−7^ M dihydrotestosterone. Before withdrawing the medium and processing the cells for mRNA extraction, cells were checked under microscope to verify their integrity. Results of gene expression studies determined by reverse-transcription quantitative PCR analyses (RT-qPCR) are presented in Figure 6. Sex hormone exposure did not affect MR (A) and *Sgk1* (D) expression in this renal cell model. While estradiol and dihydrotestosterone both increased GR, *αENaC*, *Gilz* and *Hsd11b2* mRNA levels, significant differences between estradiol and dihydrotestosterone (DHT) were observed for GR, *αENaC* and *Gilz* mRNA levels (*p* < 0.01, *p* < 0.01 and *p* < 0.05, respectively), with a 1.5- to 2-fold increase of expression with DHT, whilst estradiol only moderately modified their expression. These results confirm the influence of sex hormones on the expression pattern of players of the mineralocorticoid pathway, most notably for *Gilz* mRNA expression, that corroborates our in vivo studies. However, this sex-specificity of expression pattern of renal mineralocorticoid signaling pathway does not appear to uniquely rely on sex hormones, since *Sgk1* or *Hsd11b2* mRNA expression in this cell-based model and under such experimental conditions, is not differentially modified by androgens or estrogens.

## 3. Discussion

In the present paper, we demonstrated both in vivo and in vitro that there exists a sexual dimorphism in the expression pattern of different players of the mineralocorticoid pathway at the mRNA and protein level in the kidney. Indeed, we observed a drastic difference between male and female mice in renal expression of 11βHSD2, which regulates glucocorticoid metabolism and action, thereby conferring mineralocorticoid selectivity in aldosterone epithelial target tissues and of two regulated target genes, *Sgk1* and *Gilz*. These results were confirmed in two series of animals of different mixed genetic background, providing support for an important and well-conserved regulatory mechanism. Of interest, these different genes did not follow a similar sex-specific pattern of expression, as renal *Gilz* mRNA levels were down-regulated in females compared to males, while *Hsd11b2* and *Sgk1* expression remained higher in the kidneys of female compared to male kidneys. This result was confirmed for 11βHSD2 at the protein level. Increased expression of *Hsd11b2* in females has previously been described in the literature, but no significant increase in 11βHSD2 activity was observed in estrogen-treated rats [24]. Using LC-MSMS technology, we have assessed hormonal levels of corticosterone and 11-dehydrocorticosterone and have shown a significant difference between male and female adult mice. These novel results highlight that steroids are produced/secreted at a higher level in females. However, as the plasma ratio between these compounds is the result of equilibrium between 11βHSD2 and 11βHSD1 activities, no definite conclusion can be drawn from these results regarding differential 11βHSD2 activity between sexes. Measurements of urinary steroids in mice, in comparison to plasma steroid levels, will be much more relevant to directly assess renal 11βHSD2 activity. However, this technology is not yet at our disposal for urinary steroid measurement in mice.

Further studies, particularly in human physiology, will also need to be performed in order to assess whether 11βHSD2 sexually dimorphic expression is relevant to the differential cortisol metabolism observed between men and women [24,25].

Animals from the same mixed genetic background were used to measure basal systolic blood pressure and HR. These two cardiovascular parameters also exhibited a drastic sex dimorphism, with a significant lower basal systolic blood pressure and HR in females compared to males. Size did differ significantly between males and females (*p* < 0.001*)*, which could account in part for differential basal systolic blood pressure. However, it cannot be the unique explanation, as numerous studies demonstrated that the higher blood pressure observed in humans and male mice are linked to testosterone production rather than to an increase in weight/adiposity. Indeed, when male mice were castrated, the difference in blood pressure disappeared with regards to females, and when testosterone was given to ovariectomized female mice, their blood pressure increased, regardless of body weight [26]. Moreover, when the ratio of kidney weight over total body weight was compared between males and females in our studies (*p* = 0.15), no difference was observed, suggesting that the difference observed in the pattern of expression of renal mineralocorticoid target genes does not rely on differential body composition. 

Thus, a functional link between differences observed in gene expression in the mineralocorticoid pathway and the significant difference between blood pressure and HR from male to female could be strongly suggested. This has also been evoked by other authors considering the impact of sex on the renin–angiotensin–aldosterone system and its relation to cardiovascular diseases [6,7]. Particularly, there is accumulating evidence that cardiovascular responsiveness to the aldosterone signaling pathway may vary depending upon sex. For example, serum levels of aldosterone correlate with left ventricular hypertrophy and left ventricular mass index in women but not in men [27]. Likewise, a remarkable sexual dimorphism has been reported in several components of the hypothalamic–pituitary–adrenal axis in mice, with females displaying higher adrenal weight, plasma ACTH (Adrenocorticotropic Hormone), corticosterone, and aldosterone levels than males [28]. However, to date, clinical or experimental studies have demonstrated no or little significant sex-specific difference in cardiac MR expression or response to cardiac MR blockade [8,9,29,30]. Plasma aldosterone levels were not found to be significantly different between sexes in our studies. In any case, aldosterone levels would not be accountable for the variability in mineralocorticoid target gene expression, as both kidneys and lungs, which express MRs, *αENaC*, *Sgk1* and *Gilz* and respond to aldosterone, display different expression profiles between these two tissues in the same mouse. Additionally, as stated by Mihailidou et al. [6] elevated circulating levels of aldosterone do not always translate to a physiological response, with reduced peripheral vascular resistance and no change in blood pressure reported with enhanced aldosterone levels, suggesting that additional mechanisms are involved.

In the present study, we established for the first time that these sex differences in renal expression pattern of the mineralocorticoid pathway appear early during kidney development, which adds credit to the hypothesis of a sex difference in the developmental programming of hypertension [7]. Indeed, different animal models of adverse perinatal events, such as intra-uterine growth retardation from overexposure to glucocorticoids [19], placental insufficiency [31] or high sodium intake [32], lead to a notable sex difference in arterial blood pressure in adulthood [31,33,34,35,36,37,38] with a significant increased prevalence of hypertension in males in relation to testosterone levels [39], while estrogens exert a protective effect [40]. All of these animal models also present with early modifications in kidney organogenesis (reduced nephron number) and/or with alterations in expression of some mineralocorticoid target genes [19,41]. Of interest, sexual dimorphic expression of players of the mineralocorticoid pathway was observed in our in vivo studies in mouse fetuses and newborns as well as in adulthood, but never in 7.5-day-old mice. Given that fetuses and neonates are exposed to sex hormone secretion originating from their gonads, the placenta and the mother, and considering that puberty occurs about six weeks after birth in mice, it could be proposed that the 7.5-day postnatal stage constitutes a peculiar developmental stage in which animals are somehow protected against the influence of sex hormones, with variance in the three other developmental stages evaluated, i.e., the prenatal, the perinatal period and in adulthood. Thus, it is very likely that a direct relationship may exist between the mineralocorticoid pathway, sex hormones and their receptors. This is partly confirmed by our in vitro studies where we demonstrated that estradiol and DHT directly exert a differential effect on expression of renal mineralocorticoid target genes and on that of the *Hsd11b2*. Whether this is mediated via estrogen receptors (ERs) and androgen receptors (ARs) remains to be determined. However, several studies support this hypothesis. Indeed, experimental studies have demonstrated that estrogen and mineralocorticoid receptors are both expressed in cardiac myocytes, fibroblasts, and vascular cells [42,43]. Moreover, in rodent models, activation of ERs protects the cardiovascular system against the detrimental effects of aldosterone/salt treatment, including effects on blood pressure, cardiac hypertrophy, and vascular fibrosis [44]. Recent studies have underlined a direct functional interference between ERs and MRs, with an inhibitory effect of ERs on MR transactivation capacities by dimerization between the two nuclear receptors, specifically in endothelial cells [21]. These results suggest a protective mechanism of estrogen signaling on aldosterone-mediated vascular sensitivity to hypertension in females before menopause. Whether this interaction also occurs at the kidney level will need to be further investigated. Aside from these studies, currently very little is known about a potential effect on dimerization/cross-talk/heterologous desensitization of MRs by ARs, however, given the high sequence homology, these interactions might be possible [45,46].

An additional hypothesis to explain sex differences in *Sgk1*, *Gilz* and *Hsd11b2* mRNA levels, could be the implication of the glucocorticoid pathway and the GR. Indeed, glucocorticoids and mineralocorticoids share a similar affinity for the MR and GR. MRs may bind identical hormone response elements on genomic DNA and recruit common coregulators, and they are involved in the induced transcription of shared target genes, particularly, *Sgk1* and *Gilz* [15,47]. Both are expressed in numerous tissues, including the distal convoluted tubule and cortical collecting duct in the kidney [15].

Thus, differences observed notably in renal *Gilz* expression, which is down regulated in female kidneys, could be a direct consequence of the upregulation of renal *Hsd11b2* expression and presumably activity, thereby enhancing glucocorticoid metabolism and active glucocorticoid clearance and ultimately leading to a reduction of GR-regulated target gene expression in female kidneys. In addition, this could thereby favor an aldosterone-MR signaling pathway and a potential stimulating effect on *Sgk1*. Thus, it is likely that a balance between activation of these two pathways could be established in the adult kidney, in a sex-specific manner. A previous study has readily demonstrated a differential activation of *Sgk1* and *Gilz* between MRs and GRs in the kidney in a model of overexpression of renal GRs [48]. Moreover, there are reports indicating that GRs and ERs may also interact and modulate each other’s downstream signaling [49], and that sexually dimorphic actions of glucocorticoids exist [50]*.* Of particular interest, estrogens have been shown to antagonize the glucocorticoid induction of the *Gilz* gene [51], which is also suggested in our in vitro cell model, with downregulation of both GR and *Gilz* mRNA levels induced by estradiol. Furthermore, a sex-specific expression of co-activators and co-repressors and other nuclear receptors has been previously proposed [52].

We also showed that this sex-specific pattern of expression of the mineralocorticoid pathway has a tissue-specificity with a distinct pattern of sexual dimorphism in the lungs, known as another mineralocorticoid epithelial target tissue implicated in ion exchange and electrolyte and fluid balance through regulated expression of similar target genes: *ENaC*, *Sgk1* and *Gilz* for instance. As we have previously demonstrated, these two mineralocorticoid target tissues do not exhibit similar expression patterns for the different players of the mineralocorticoid pathway during development, with a constant expression during the perinatal period in the lung and downregulation in the kidney [22]. Thus, it was expected that these two tissues might behave differentially with regards to sex. Indeed, in contrast to the kidney, pulmonary *Gilz* mRNA expression, for instance, is upregulated to a greater degree in females compared to males. Recent studies have underlined sex differences in respiratory function during lung development and in adulthood [3,53,54,55]. Moreover, female smokers have an increased risk of chronic obstructive pulmonary disease in comparison to male smokers with similar history of cigarette smoke exposure, both in human and mice; however, the underlying mechanisms are still under investigation [56,57]. Thus, the mineralocorticoid signaling pathway, whose role is crucial in lungs particularly during the neonatal period [22,58,59], could participate to this sexual dimorphism.

In conclusion, we have demonstrated a tissue-specific, sex-dependent and developmentally regulated pattern of expression of the mineralocorticoid pathway that could have important implication in physiology and pathology. Indeed, early events such as prematurity or growth restriction might alter or exacerbate the programming of such patterns of expression and induce hypertension, most particularly in males [60]. Better knowledge of the mechanisms underlying such sex differences in mineralocorticoid-signaling gene expression and their related control of arterial blood pressure should bring new insights into the molecular determinants of hypertension and will impact therapeutic strategies, with new sex-targeted approaches to prevent cardiovascular and renal diseases.

## 4. Materials and Methods

### 4.1. Mouse Samples

Wild-type mouse kidneys and lungs were collected at different developmental stages from 17.5 days of gestation (E17.5) to 3 postnatal months of age (M3) from female and male mice of mixed background (B6D2F1). For each animal, one kidney and one lung were snap-frozen in liquid nitrogen for RT-qPCR analyses or immunoblotting. Blood samples were also collected on EDTA(Ethylenediaminetetraacetic acid)-containing tubes after sacrifice, and processed for 11-dehydrocorticosterone and corticosterone measurements by LC-MSMS [61]. Results obtained at M3 were confirmed in another independent series of experiments with wild-type mice originating from mixed background of different strains (C57B6/129S). At least 6 female mice and 6 male mice were sacrificed for each developmental stage and each experiment. Mice were housed and handled according to the National Institute of Health Guidelines. The study is part of an approved project by the ethics committee CEEA 26 (#2012_021).

### 4.2. Blood Pressure Measurements

Blood pressure measurements were conducted in the animal facility of the FRIM (Fédération de Recherche en Imagerie Multi-Modalité, Paris Diderot University, France). Animals were acclimatized to the facility for at least 5 days, and the first two days of blood pressure measurements were not included. Systolic blood pressure (SBP) was measured by tail-cuff plethysmography in trained animals as previously described [62]. Briefly, mice were restrained in a clear, plastic tube at room temperature, and the cuff was placed on the tail and inflated to 200 mmHg. The reappearance of a pulse during deflation of the cuff was used to determine SBP. To minimize stress, no animal was restrained for more than 10 min at a time, and a minimum of six clear SBP recordings were taken per animal. Heart rate was derived from the pulse to pulse interval.

Nine female mice and eleven male mice aged 6 months were used for the analysis. Results are expressed as the mean ± SEM of at least six measurements of systolic blood pressure and HR for each mouse of each sex per day over three consecutive days.

### 4.3. Reverse Transcription Quantitative PCR

Total RNA was extracted from tissues or cells with the TRIZOL reagent (Life Technologies, Villebon-sur-Yvette, France) according to the manufacturer’s recommendations, and RNA was thereafter processed for RT-qPCR, as previously described [18]. Total RNA (1 μg) isolated from frozen samples, was subjected to deoxyribonuclease I Amplification Grade treatment (Biolabs, Evry, France) and then reverse transcribed by use of High-Capacity cDNA reverse transcription kit from Applied Biosystems (Life Technologies). Samples were diluted 10-fold after which 1/20 of the reverse transcription reaction was used for reverse transcription quantitative PCR (RT-qPCR) using the Fast SYBR Green Master Mix (Applied Biosystems) containing 300 nM of specific primers. RT-qPCR was carried out on a StepOnePlus Real-Time PCR System (Applied Biosystems). Reaction parameters were as follows: 95 °C for 20 s, then 40 cycles at 95 °C for 1 s and 60 °C for 20 s. For standards preparation, amplicons were subcloned into pGEMT-Easy plasmid (Promega, Charbonnières-les-Bains, France) and sequenced to confirm the identity of each sequence. Standard curves were generated using serial dilutions of linearized standard plasmids, spanning six orders of magnitude. Samples were amplified in duplicate or triplicate. Ribosomal 18s RNA was used as an internal control for data normalization (for mouse experiments, as other housekeeping genes may vary upon renal developmental stages), and *36b4* mRNA in cell culture experiments. Relative expression of a given gene is expressed as the ratio of attomoles of specific gene per femtomole of 18s or per attomoles of housekeeping gene (*36b4*). All experiments were performed in triplicate from two or three independent reverse transcriptions. Thus, final results represent the relative expression normalized to that obtained in samples from male mice at D0 (Figure 2), or samples from male mice at M3 (Figure 3 and Figure 5) or samples incubated with vehicle (ethanol) (Figure 6) which was each time arbitrarily set at 1. Primer sequences of genes analyzed by RT-qPCR were previously published [18].

### 4.4. Western Blot Analyses

Total protein extracts were prepared from frozen male and female murine kidneys, and subsequently processed for multiplex detection of 11βHSD2 protein together with α-tubulin protein for loading normalization. Immunoblots were incubated overnight in 5% milk/Tris-buffered saline/0.1% Tween 20 with rabbit anti-11βHSD2 (1:1000, Santa Cruz SC-20176, Heidelberg, Germany) and mouse anti-tubulin antibodies (1:5000 Sigma, Saint-Quentin-Fallavier, France) followed by incubation for 1 h at room temperature with secondary antibodies coupled to a fluorochrome, Dylight anti-Rabbit 800 at a dilution of 1:10,000 or Dylight anti-Mouse 680 at a dilution of 1:15,000 (Fischer Scientific, Ilkirch, France). Detection and quantification of specific fluorescent signals was performed in multiplex using an Odyssey Fc (LI-COR, Lincoln, NE, USA).

### 4.5. Cell Culture

KC3AC1 cells were seeded on collagen I-coated 24-well plates (Collagen I from Institut Jacques Boy, Reims, France), and routinely cultured as previously described [23] for 7 days at 37 °C in a humidified incubator gassed with 5% CO_2_ within an epithelial medium. To study sex hormone actions, the epithelial medium was replaced after day 7 of culture by a minimal medium (MM), which has the same composition as the epithelial medium, but which lacks dexamethasone and dextran charcoal-treated serum. Ethanol only (control) or estradiol or dihydrotestosterone (Acros Organics, Noisy Le Grand, France) at a concentration of 10^−7^ M was added to the medium for 24 h. Cells were then harvested and mRNA was extracted.

### 4.6. Statistical Analyses

Results are expressed as mean ± SEM of at least three independent analyses with at least six samples for each sex at each developmental stage or each experimental condition. Statistical analyses were performed using a nonparametric Mann Whitney *t*-test to compare two parameters and nonparametric Kruskall–Wallis multivariance analyses to compare three independent parameters or more (Graphpad Prism 5, Graphpad Software, Inc., San Diego, CA, USA), with significant threshold set at *p*-value < 0.05.
